# Limiting viral replication in hematopoietic cells delays Rift Valley fever virus disease progression in C57BL/6 mice

**DOI:** 10.1128/jvi.01261-25

**Published:** 2025-09-08

**Authors:** Lingqing Xu, Alden C. Paine, Dominique J. Barbeau, William Klimstra, Anita K. McElroy

**Affiliations:** 1 Division of Pediatric Infectious Disease, Department of Pediatrics, University of Pittsburgh School of Medicine 12317 , Pittsburgh, Pennsylvania, USA; 2 Center for Vaccine Research, University of Pittsburgh 588296, Pittsburgh, Pennsylvania, USA; 3 Department of Immunology, University of Pittsburgh 215866, Pittsburgh, Pennsylvania, USA; University of Kentucky College of Medicine, Lexington, Kentucky, USA

**Keywords:** Rift Valley fever virus, viral pathogenesis, hematopoietic cells, type I IFN, microRNA

## Abstract

Rift Valley fever virus (RVFV) causes mild to severe disease in livestock and humans. It was first identified in 1931 during an epizootic in Kenya and has spread across Africa and into the Middle East. Hematopoietic cells are one of the major targets of RVFV *in vivo*; however, their contribution to RVFV pathogenesis remains poorly understood. To address this, we generated a recombinant miRNA-targeted virus, RVFVmiR-142, to limit viral replication in hematopoietic cells and evaluated the consequences on RVFV disease manifestations in C57BL/6 mice. MicroRNAs are evolutionarily conserved non-coding RNAs that regulate mRNA expression. RVFVmiR-142 includes an insertion of four repeated sequences targeted by hematopoietic-specific miRNA-142. RVFVmiR-MM, which contains four repeats of sequences that are not targets of any known miRNA, was generated as a control. RVFVmiR-142 showed restricted replication *in vitro* compared to RVFVmiR-MM. C57BL/6 mice infected with 2 TCID_50_ of RVFVmiR-142 had delayed disease progression vs RVFVmiR-MM-infected mice, the phenotype of which was overcome by higher infection doses. The difference in disease progression at low dose was eliminated in *MiR-142* KO mice, confirming the specificity of the phenotype. A timed euthanasia study showed delayed viral replication of RVFVmiR-142 compared to RVFVmiR-MM in infected mice, most notably in tissues largely composed of hematopoietic cells. Furthermore, control of RVFVmiR-142 replication in the popliteal lymph nodes correlated with an increased type I IFN response, which was lacking in the liver tissue where RVFVmiR-142 replication continued to increase. These data suggest that RVFV replication in hematopoietic cells contributes to viral amplification and/or spread.

**IMPORTANCE:**

RVFV is a segmented, single-stranded, negative-sense RNA virus vectored by diverse genera of mosquitoes and can infect a variety of wild animals, domesticated livestock, and humans. Despite the increase in both the range and frequency of RVFV outbreaks over the years, there is currently no vaccine or treatment available for human use against RVFV. Mononuclear phagocytic cells (MPCs) are one of the major targets of RVFV *in vivo*; however, the contribution of RVFV replication in these cells to its pathogenesis has not been well characterized. In this study, we generated a recombinant miRNA-targeted virus with restricted replication in hematopoietic cells and examined its pathogenesis in C57BL/6 mice. This study demonstrates that hematopoietic cell infection contributes to viral pathogenesis by augmenting viral amplification and/or spread.

## INTRODUCTION

Rift Valley fever virus (RVFV) is an important hemorrhagic fever virus that belongs to the *Phlebovirus* genus in the family *Phenuiviridae* in the *Bunyaviricetes* class. It is listed by the National Institute of Allergy and Infectious Diseases (NIAID) as a category A high-priority pathogen and classified as a select agent by both U.S. Department of Health and Human Services (DHHS) and U.S. Department of Agriculture (USDA). Over the last two decades, RVFV has expanded its transmission events across and outside of Africa at an increasing frequency, necessitating the urgency to better understand this emerging virus (1–3).

Mosquitoes are vectors of RVFV. In regions where epizootics have occurred, RVFV has been isolated from more than 53 species in 8 genera of mosquitos, among which *Aedes* and *Culex* are considered the main vectors (4). RVFV primarily affects domestic animals including sheep, goats, cows, and camels, with pregnant and newborn ruminants being the most susceptible (5, 6). Humans become infected with RVFV through mosquito bites or contact with the bodily fluids or tissues of infected animals. Most infected humans develop a self-limiting febrile illness and often recover within a week. A small percentage of individuals will develop more severe disease including hepatitis, ocular disease, meningoencephalitis, or hemorrhagic fever, often with high mortality rates (7). However, the determinants for severe outcome remain poorly understood. To date, there have been no licensed vaccines or treatments for use against RVFV in humans, highlighting the importance of studying the pathogenesis of this virus so that its clinical consequences can be minimized.

The lipoprotein receptor-related protein 1 (mouse Lrp1/human LRP1) was recently identified as a host entry factor for RVFV (8). Consistent with Lrp1 expression, mononuclear phagocytic cells (MPCs), hepatocytes, and neurons are all major RVFV targets *in vivo* (8–10). To study how each of these cell types contributes to host immunity and RVFV pathogenesis, we took advantage of tissue-specific miRNAs to restrict viral replication in cells of interest (11–15). MicroRNAs are a family of small single-stranded RNAs that play essential roles in developmental and other biological processes (16). They are 21–23 nucleotides long and bind to target mRNAs to induce mRNA degradation or translation inhibition, therefore regulating gene expression (16). Importantly, many miRNAs are evolutionarily conserved and cell-type specific (17). Our lab recently demonstrated that using miR-122 targets to limit hepatic replication of RVFV led to altered disease manifestations in C57BL/6 mice with a switch from acute hepatitis to late-onset encephalitis (18). In this study, the role of viral replication in hematopoietic cells in RVFV pathogenesis is examined.

Cells of hematopoietic origin, including myeloid and lymphoid, play key roles in regulating both the innate and adaptive immune responses during viral infection (19, 20). Single-nucleotide polymorphisms in multiple genes encoding innate immune receptors and signaling mediators, including interferon-alpha/beta receptor (IFNAR), correlate with Rift Valley fever disease phenotypes in humans (21). Previous research has shown that transgene expression from vectors incorporating miR-142 target sequences was effectively suppressed in hematopoietic lineages and maintained in non-hematopoietic cells, highlighting the specific expression pattern of miRNA-142 in cells of the hematopoietic system, which was confirmed by small RNA library sequencing in later studies (22, 23). Therefore, miR-142 target sequences were selected for incorporation into the S segment of RVFV genome to effect hematopoietic-specific restriction of viral replication. RVFV has a tripartite genome consisting of three segments: S, M, and L. The L segment encodes the RNA-dependent RNA polymerase required for genome replication and transcription (24). The M segment encodes two envelope glycoproteins, Gn and Gc, and two non-structural proteins, NSm and the 78 kDa protein (25). The S segment encodes nucleoprotein N and a non-structural protein NSs, which is the major virulence factor for RVFV infection via antagonizing IFN-β expression (26, 27).

By comparing *in vivo* pathogenesis of hematopoietic restricted vs control RVFV, a delayed disease progression phenotype for RVFV in C57BL/6 mice was observed when viral replication in hematopoietic cells was restricted. Investigation of host immunity demonstrated an association between local control of viral replication and type I IFN expression in a hematopoietic cell dominant tissue, the popliteal lymph node (PLN). In contrast, in a non-hematopoietic cell dominant tissue, the liver, we did not observe an activation of type I IFN response and the viral replication of RVFVmiR-142 continued to increase over time. These results demonstrate the contribution of RVFV replication in hematopoietic cells to RVFV *in vivo* pathogenesis.

## RESULTS

### Recombinant RVFVmiR-142 showed restricted replication *in vitro*

**Fig 1:**
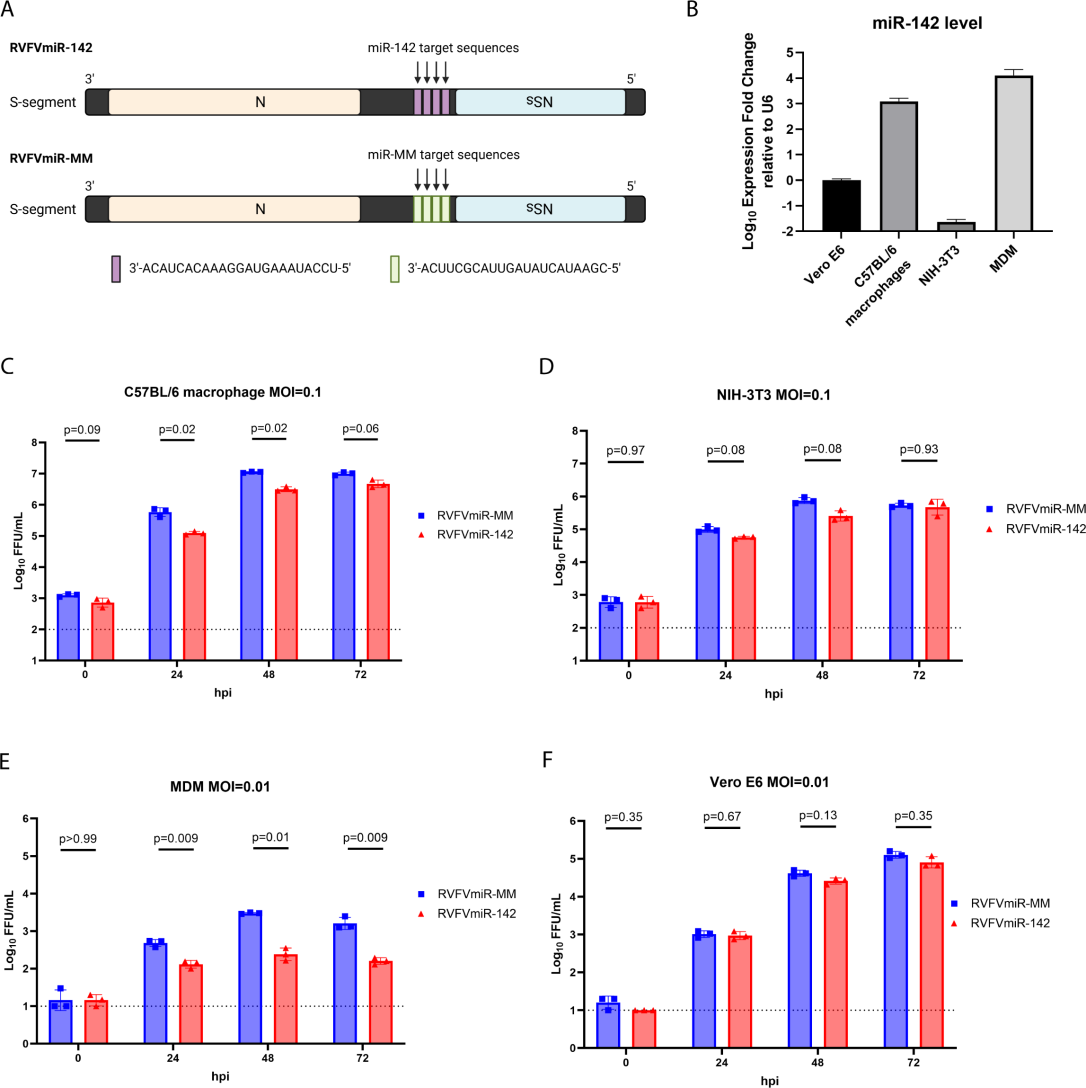
RVFVmiR-142 showed restricted replication in cells expressing high levels of miR-142 compared to control RVFVmiR-MM. (**A**) Schematic design of RVFVmiR-142 and RVFVmiR-MM. Graph was created in BioRender. (**B**) RNA extracted from triplicates of Vero E6 cells, C57BL/6 macrophages, NIH-3T3 cells, or primary human monocyte-derived macrophages (MDM) was used for reverse transcription followed by real-time PCR targeting either miR-142 or U6 gene as control. Expression fold change of miR-142 relative to U6 was normalized to that of Vero E6 cells. (**C–F**) Triplicates of C57BL/6 macrophages (**C**) or NIH-3T3 cells (**D**) were infected with RVFVmiR-142 or RVFVmiR-MM at MOI of 0.1. Triplicates of MDM (**E**) or Vero E6 cells (**F**) were infected with RVFVmiR-142 or RVFVmiR-MM at MOI of 0.01. Supernatants were collected at various time points post-infection and analyzed by focus-forming unit (FFU) assay. Data were analyzed using multiple lognormal *t* tests for comparison of viral titers at each time point and *P* values are shown. The dotted line indicates the limit of detection (LOD) of FFU assay.

miR-142 is preferentially and highly expressed in the hematopoietic system and is conserved in vertebrates (23). Therefore, to study the relevance of viral replication in hematopoietic cells to RVFV pathogenesis, a recombinant virus (RVFVmiR-142) was generated which incorporated four repeats of miR-142 target sequences into the negative strand of RVFV S segment. A control virus, RVFVmiR-MM, was also generated which contains an insertion of four repeats of sequences that are not targeted by any known miRNA described in the miRNA database (mirbase.org) (Fig. 1A). To demonstrate hematopoietic restriction of RVFVmiR-142 *in vitro*, we infected immortalized C57BL/6 mouse macrophages and primary human monocyte-derived macrophages (MDMs), both of which express high levels of miR-142, with either RVFVmiR-142 or RVFVmiR-MM. NIH-3T3, a mouse fibroblast cell line, and Vero E6, an epithelial cell line isolated from the kidney of an African green monkey, were used as controls due to their absence or low expression level of miR-142 (Fig. 1B). C57BL/6 macrophages and NIH-3T3 cells were infected with virus at a multiplicity of infection (MOI) of 0.1. However, this MOI caused rapid cell death in human MDMs; therefore, an MOI of 0.01 was used to infect MDMs and Vero E6 cells. Supernatants were collected at various times post-infection, and viral titers were measured. As expected, these two viruses had no difference in replication in NIH-3T3 or Vero E6 cells over time (Fig. 1D and F). In contrast, RVFVmiR-142 had restricted replication compared to RVFVmiR-MM in both C57BL/6 mouse macrophages and human MDMs, which was notable as early as 24 h post-infection (hpi) (Fig. 1C and E). The difference in viral replication between RVFVmiR-142 and RVFVmiR-MM was diminished at 72 hpi in C57BL/6 macrophages, probably due to increased cell death in RVFVmiR-MM-infected cells. The greater restriction of RVFVmiR-142 in human MDMs compared to that in C57BL/6 mouse macrophages was likely due to a 10-fold higher expression level of miR-142 in MDMs (Fig. 1B). Previous studies have shown that the expression of some miRNAs is altered during viral infection (28). Due to the design of our recombinant viruses, assays quantifying levels of miRNA will also detect sequences of transcribed (+) sense viral RNA, therefore we were unable to differentiate between endogenous miR-142 and (+) viral RNA in RVFVmiR-142 infected samples. As an alternative, we examined the level of miR-142 in cells infected with recombinant RVFV ZH501 or RVFVmiR-MM. RVFV ZH501 is a wild-type RVFV strain isolated from an acutely and fatally infected human case at the Zagazig Hospital (ZH) during the 1977 outbreak in Egypt (29). There was a slight increase of miR-142 level in RVFV ZH501 infected C57BL/6 macrophages at 48 hpi compared to that at 0 hpi. Importantly, we did not observe any difference in miR-142 level in RVFVmiR-MM infected cells at 48 hpi vs 0 hpi, suggesting that the restricted replication of RVFVmiR-142 compared to that of RVFVmiR-MM was not due to a change in the expression level of miR-142 post-infection (Fig. S2). Together, these data demonstrate restricted replication of RVFVmiR-142 in hematopoietic cells of both mouse and human origin in which miR-142 is highly expressed.

### RVFVmiR-142-infected C57BL/6 mice had delayed disease and increased survival compared to RVFVmiR-MM-infected mice, and this phenotype required the presence of miR-142

**Fig 2:**
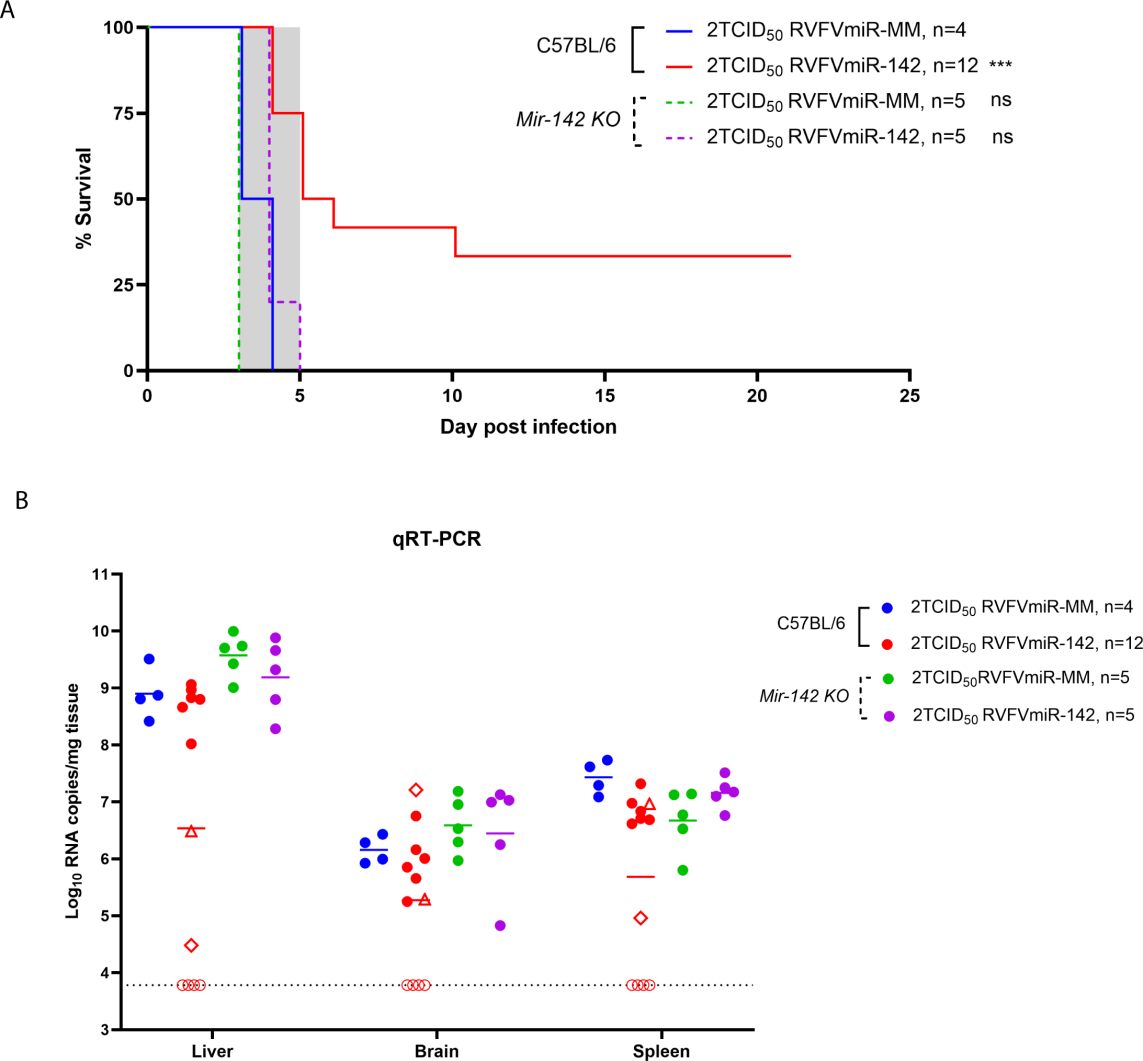
Increased survival and delayed disease progression were observed in C57BL/6 mice infected with RVFVmiR-142. (**A**) C57BL/6 mice or *Mir-142 KO* mice were monitored for survival after footpad infection with 2 TCID_50_ of either RVFVmiR-MM or RVFVmiR-142. Log-rank (Mantel-Cox) test was performed to compare survival curves of each group to control C57BL/6 group infected with RVFVmiR-MM. ***P < 0.001, ns, non-significant. Shaded gray area represents the normal range of time to death (3–5 dpi) in C57BL/6 mice after recombinant RVFV ZH501 infection. (**B**) Viral RNA loads of RVFV L segment in the liver, brain, and spleen tissues from mice at the time of death or euthanasia. A dotted line indicates limit of detection (LOD) of the qRT-PCR assay. A short solid line in each group represents the geometric mean. Open circles in red represent mice that survived until the end of the experiment (21 dpi). Open triangle in red represents a RVFVmiR-142-infected mouse that succumbed to disease at 6 dpi, and open diamond in red represents a RVFVmiR-142-infected mouse that required euthanization at 10 dpi.

To examine the consequences of hematopoietic-restricted replication of RVFV *in vivo*, we infected C57BL/6 mice with either RVFVmiR-142 or RVFVmiR-MM at a dose of 2 TCID_50_. C57BL/6 mice infected with 2 TCID_50_ of recombinant RVFV ZH501 consistently succumb to acute hepatitis between 3 and 5 days post-infection (dpi) (30). Even though RVFVmiR-MM showed some attenuation *in vitro* compared to recombinant RVFV ZH501 (Fig. S1), mice infected with RVFVmiR-MM developed typical RVFV hepatic disease and succumbed to disease early (Fig. 2A, blue line). In contrast, mice infected with RVFVmiR-142 showed a mixed phenotype; half of the infected mice died or required euthanasia between 4 and 5 dpi, while the other half had either delayed disease progression or survived until the end of the experiment without showing clinical signs (Fig. 2A, red line). Because 2 TCID_50_ is a low infection dose, seroconversion was demonstrated via ELISA using serum of all surviving mice at the end of the experiment to confirm the infection. Additionally, sanger sequencing of RNA extracted from liver tissues of RVFVmiR-142-infected mice that succumbed to disease early (4 and 5 dpi) confirmed the retainment of miR-142 target sequences in the viral genome, ruling out the possibility of a genetic revertant. Results from qRT-PCR performed at the time of euthanasia demonstrated a relationship between viral RNA loads and the timeline of disease progression. RVFVmiR-142-infected mice that died early had the highest viral RNA loads in the liver, about 3-logs lower viral RNA loads in the brain, and 2-logs lower viral RNA loads in the spleen (Fig. 2B, closed red circles), similar to control mice infected with RVFVmiR-MM (Fig. 2B, closed blue circles). In contrast, RVFVmiR-142-infected mice that survived until the end of the experiment had viral RNA loads around or below the limit of detection (LOD) in all tissues examined (Fig. 2B, open red circles). Notably, one mouse that required euthanasia at 10 dpi (Fig. 2B, open diamond) had viral RNA loads low in the liver and high in the brain, suggesting viral clearance from the liver and invasion into the brain. The other mouse that succumbed to disease at 6 dpi (Fig. 2B, open triangle) had relatively high level of viral RNA in the liver and low level of viral RNA in the brain, suggesting the disease manifestation in that mouse was still hepatic. To examine if the delayed disease progression and increased survival observed in RVFVmiR-142-infected mice was specific to the expression of miR-142, *MiR-142 KO* mice were infected with either RVFVmiR-MM or RVFVmiR-142 at a dose of 2 TCID_50_. Both groups of mice died or required euthanasia between 3 and 5 dpi, and their viral RNA loads were highest in the liver (Fig. 2A and B, green and purple). These results demonstrate that the change in disease manifestation in mice infected with RVFVmiR-142 was dependent on the expression of miR-142, confirming the specificity of the phenotype.

### Viral replication and spread were delayed in RVFVmiR-142-infected mice

**Fig 3:**
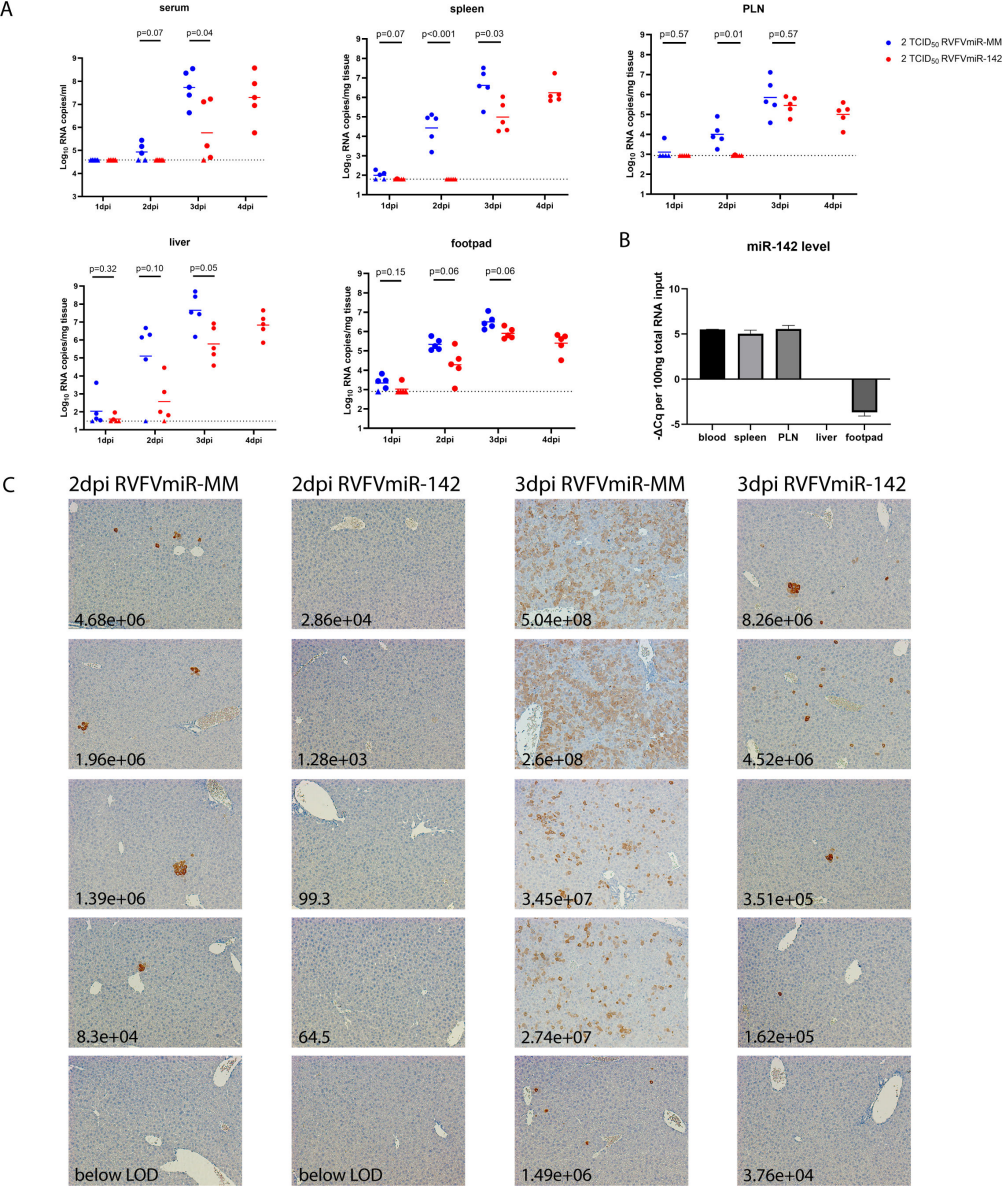
Viral replication and spread of RVFVmiR-142 was delayed *in vivo* compared to RVFVmiR-MM. Groups of mice (*n* = 5) infected with either RVFVmiR-MM or RVFVmiR-142 at 2 TCID_50_ were euthanized at 1, 2, 3, and 4 dpi. (**A**) Viral RNA loads in the serum, liver, spleen, footpad, and popliteal lymph node (PLN) from mice at the time of euthanasia. A dotted line indicates limit of detection (LOD) of the qRT-PCR assay. Short solid line in each group represents geometric mean. Triangles represent data points below LOD. Statistical analysis was performed using multiple lognormal *t* tests, and *P* values were shown. (**B**) One hundred nanograms of RNA extracted from various tissues of C57BL/6 mice was used for reverse transcription followed by real-time PCR targeting miR-142. Level of miR-142 in each tissue was normalized to that in the liver and plotted as the difference of Cq value per 100 ng total RNA. (**C**) Examples of liver tissue staining by IHC probing for RVFV N protein imaged with 10× objective. Each column includes five representative images from individual mice infected with either RVFVmiR-MM or RVFVmiR-142 at 2 or 3 dpi. The vertical order of images in each group corresponds to the vertical order of dots shown in the liver viral RNA loads panel in (**A**). The number on each image is the viral RNA load measured in the liver tissue for that mouse.

To compare the disease progression in C57BL/6 mice infected with RVFVmiR-142 vs control RVFVmiR-MM, a timed euthanasia study was performed in which groups of five mice were infected with either of the two viruses at a dose of 2 TCID_50_ and euthanized at 1, 2, 3, or 4 dpi. As all mice infected with 2 TCID_50_ of RVFVmiR-MM in previous experiments died by 4 dpi, only RVFVmiR142-infected mice were sampled at 4 dpi in this experiment. Interestingly, a trend of delayed viral replication was noted in all tissues for RVFVmiR-142. This was most striking and statistically significant at 2 and/or 3 dpi and in samples known to have a high frequency of hematopoietic cells, namely, the serum (from whole blood), spleen, and popliteal lymph node (PLN) (Fig. 3A, first row). Relative expression of miR-142 in respective tissues is shown in Fig. 3B. To confirm the delayed viral replication of RVFVmiR-142, liver tissues were analyzed by immunohistochemistry (IHC) for RVFV antigen at 2 and 3 dpi. Overall, the extent of viral spread in the liver of each mouse correlated with the viral RNA load in the liver (Fig. 3C). At 2 dpi, 1 out of 5 mice in the RVFVmiR-MM-infected group had viral RNA loads below the LOD which correlated with negative antigen staining in the respective liver tissue. The remaining four mice in this group all had detectable foci with positive antigen staining in the liver (Fig. 3C, first column). In contrast, no mice in the RVFVmiR-142-infected group showed viral antigen staining in the liver at 2 dpi, consistent with low levels of viral RNA in the liver in this group (Fig. 3C, second column). At 3 dpi, viral RNA loads in the liver of RVFVmiR-MM-infected mice increased to a geometric mean of 4.29*e* + 07 copies/mg tissue. Correspondingly, antigen staining in the liver tissues became much more diffuse, reflecting rapid spread of RVFVmiR-MM (Fig. 3C, third column). In contrast, levels of viral RNA in the liver of RVFVmiR-142-infected mice were 2-logs lower with a geometric mean of 5.85*e* + 05 copies/mg tissue, and the antigen staining was localized to scattered foci (Fig. 3C, last column), reflecting the delayed progression of disease noted in mice infected with RVFVmiR-142.

### Restricted viral replication of RVFVmiR-142 in the popliteal lymph node was associated with higher levels of type I IFN-mediated response

**Fig 4:**
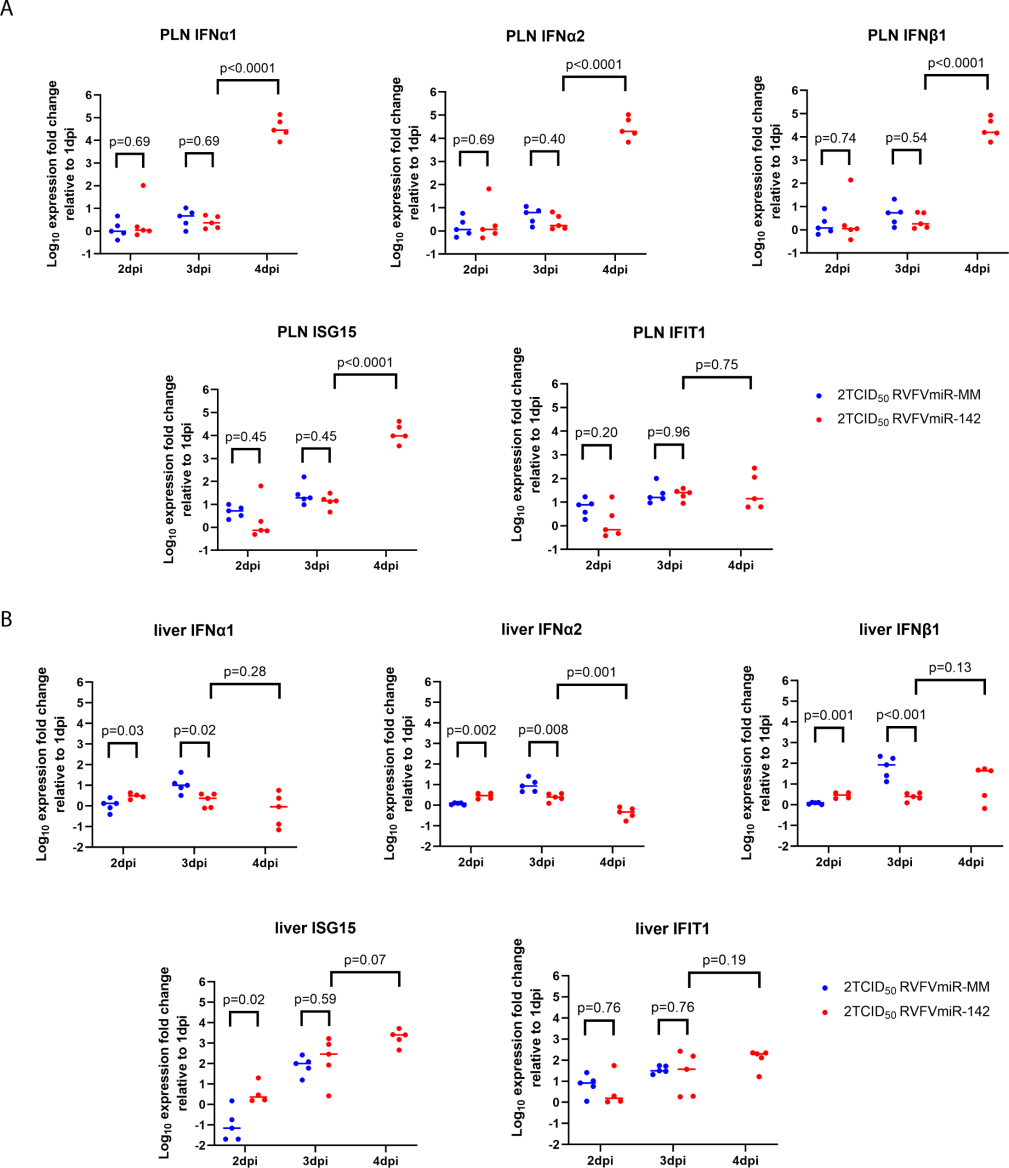
High levels of type I IFN-mediated response were observed at 4 dpi in the PLN, not in the liver, in RVFVmiR-142-infected mice. Levels of type I IFNs and two downstream ISG genes in the PLN (**A**) and liver (**B**) of mice euthanized at 1, 2, 3, or 4 dpi were measured by reverse transcription followed by qPCR and normalized to either CD45 (for PLN) or GAPDH (for liver) as control gene. Expression fold changes at 2, 3, and 4 dpi relative to 1 dpi are graphed. A short solid line in each group represents geometric mean. Statistical analysis was performed using multiple lognormal *t* tests, and *P* values were shown as indicated.

Although a delay in viral replication was observed in all tissues in RVFVmiR-142-infected mice, viral RNA loads continued to increase by 4 dpi in the serum, spleen, and liver. In contrast, viral RNA loads either stabilized or declined in the PLN and footpad of RVFVmiR-142-infected mice by 4 dpi compared to those in RVFVmiR-142-infected mice at 3 dpi, suggesting a local control of viral replication in these tissues (Fig. 3A). To examine if this controlled viral replication correlated with the innate immune response, we measured the levels of type I IFNs including IFN-α1, IFN-α2, and IFN-β, and two downstream interferon-stimulated genes (ISGs), ISG15 and interferon-induced proteins with tetratricopeptide repeats 1 (IFIT1), in the PLN of infected mice from the timed euthanasia study. A difference in the levels of these genes was not observed between mice infected with RVFVmiR-142 and RVFVmiR-MM at 2 or 3 dpi in the PLN. However, a dramatic increase in the expression of IFN-α1, IFN-α2, IFN-β, and ISG15 genes was noted in PLN at 4 dpi in RVFVmiR-142-infected mice, which correlated with the controlled viral replication in the PLN at this time point (Fig. 4A). In contrast to PLN, in the liver tissue where viral replication of RVFVmiR-142 continued to increase by 4 dpi, we did not observe an increase in type I IFN response (Fig. 4B), suggesting that the IFN response could have contributed to local immune control of viral replication for RVFVmiR-142 in the PLN, possibly due to the high percentage of residing hematopoietic cells. However, it was likely too late to prevent viral spread and replication in other tissues where viral RNA had already reached higher titers by 3 dpi (Fig. 3A).

### Delayed disease phenotype observed in mice infected with RVFVmiR-142 was eliminated at higher infection doses

**Fig 5:**
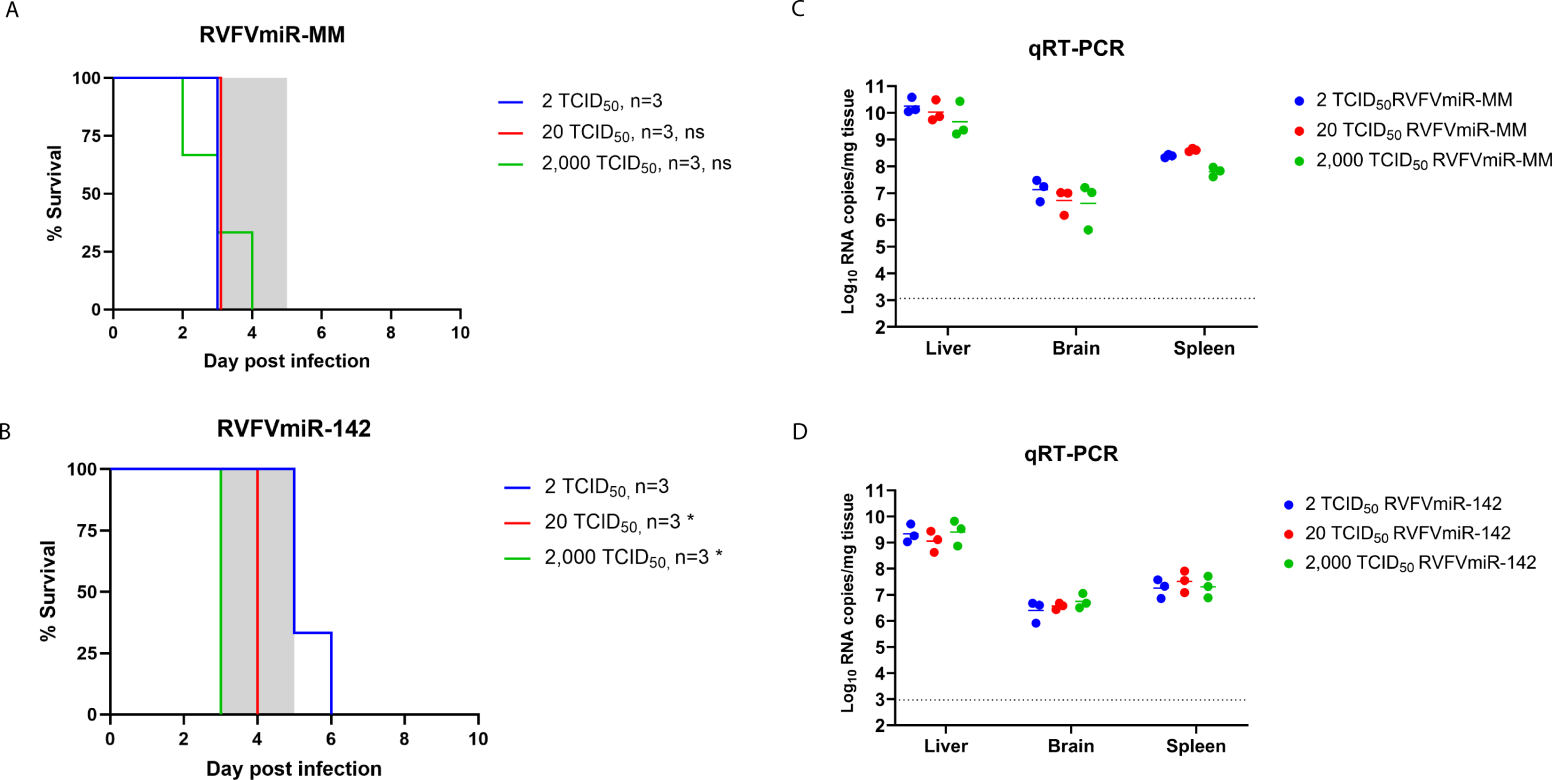
Higher doses of RVFVmiR-142 reversed the delayed disease phenotype observed at 2 TCID_50_ in C57BL/6 mice. (**A and B**) C57BL/6 mice were monitored for survival after footpad infection with either RVFVmiR-MM (**A**) or RVFVmiR-142 (**B**) at different doses. A number of mice in each group are indicated. Log-rank (Mantel-Cox) test was performed for statistical analysis on RVFVmiR-MM and RVFVmiR-142-infected groups at each dose to that of the respective 2 TCID_50_ group. ns, non-significant. *P < 0.05. Shaded gray area represents the normal range of time to death (3–5 dpi) in C57BL/6 mice after recombinant RVFV ZH501 infection. (**C and D**) Viral RNA loads in the liver, brain, and spleen tissues from mice at the time of death or euthanasia after infection with RVFVmiR-MM (**C**) or RVFVmiR-142 (**D**). A dotted line indicates limit of detection (LOD) of the qRT-PCR assay. Short solid line in each group represents geometric mean.

A prolonged survival time and increased survival in RVFVmiR-142-infected mice was noted; it was hypothesized that higher infection doses of RVFVmiR-142 would overwhelm the phenotype of RVFVmiR-142 *in vivo* and reverse the disease delay, leading to early death of infected mice. To test this hypothesis, mice were infected with either RVFVmiR-MM or RVFVmiR-142 at 2, 20, or 2,000 TCID_50_. Each dose of virus was backtitered to ensure the intended inoculum was given to each group of mice. All control mice infected with RVFVmiR-MM succumbed to disease early as expected regardless of dose, with viral RNA loads highest in the liver but also present in the spleen and brain (Fig. 5A and C). Mice infected with RVFVmiR-142 at 20 or 2,000 TCID_50_ all died or required euthanasia between 3 and 4 dpi (Fig. 5B). The delayed disease onset was only observed in mice infected with RVFVmiR-142 at a dose of 2 TCID_50_. Viral RNA loads were similar in various tissues of RVFVmiR-142-infected mice at different doses (Fig. 5D). These results support the hypothesis that higher doses of RVFVmiR-142 would overcome the delayed disease phenotype observed at 2 TCID_50_, possibly because using higher doses of virus increased the chance of non-hematopoietic cells getting infected at the injection site. An alternative possibility is that an insufficient amount of miR-142 was present in hematopoietic cells to control the virus when RVFVmiR-142 was administered at higher doses although this seems less likely given the very high quantities of miR-142 found in hematopoietic cells (23).

## DISCUSSION

MPCs, hepatocytes, and neurons have been identified as major targets of RVFV (9). However, little is known about how infection of these various cell types contributes to the overall morbidity and mortality associated with RVFV infection. To tackle this, recombinant RVFV with incorporations of sequences targeted by miRNAs selectively expressed by specific types of cells was utilized. This technique was previously used to study other viruses including Influenza A, Dengue, Vesicular Stomatitis Virus (VSV), and Chikungunya (11–14). Our lab recently demonstrated an altered disease manifestation from acute hepatitis to late-onset central nervous system (CNS) disease when viral replication was restricted in hepatocytes using a hepatocyte-specific recombinant RVFVmiR-122 (18). This approach was validated by similar findings in a study showing that knockout of the host entry factor Lrp1 in hepatocytes led to diminished RVFV replication in the liver, increased survival time, and altered clinical signs toward CNS disease in the mice (31).

In this study, recombinant RVFVmiR-142 was generated and characterized to understand the effect of hematopoietic restriction of viral replication on RVFV pathogenesis. A mixed disease phenotype was observed. Fifty percent of C57BL/6 mice infected with RVFVmiR-142 still succumbed to disease between 4 and 5 dpi, which was similar to control mice infected with RVFVmiR-MM. Previous studies have shown that miRNA-targeted viruses can escape from miRNA-mediated suppression via the deletion of miRNA targets, sometimes including the viral genome between inserted miRNA targets (15). Sanger sequencing of RNA extracted from liver tissues of RVFVmiR-142-infected mice that succumbed early confirmed that the miR-142 target sequences were still present in the viral genome, ruling out reversion of the genetic modifications. On the other hand, the remaining 50% of RVFVmiR-142-infected mice had delayed disease onset and/or increased survival. Such change in disease manifestation was eliminated in *MiR-142 KO* mice, confirming that this phenotype is due to the expression of miR-142. Interestingly, some viruses have capitalized upon the existence of miR-142 in hematopoietic cells. EEEV has four miR-142-binding sites naturally present in the viral genome (32). This interaction limited viral replication of EEEV in myeloid cells and suppressed innate immune responses, leading to increased neurovirulence (32, 33). Unlike EEEV, RVFV is a hepatotropic virus, and infection of the liver proceeds infection of the CNS; therefore, hematopoietic restriction would not necessarily be expected to be associated with neurovirulence. In fact, while delayed disease was observed with RVFVmir-142, the disease manifestation was still largely hepatic.

**Fig 6:**
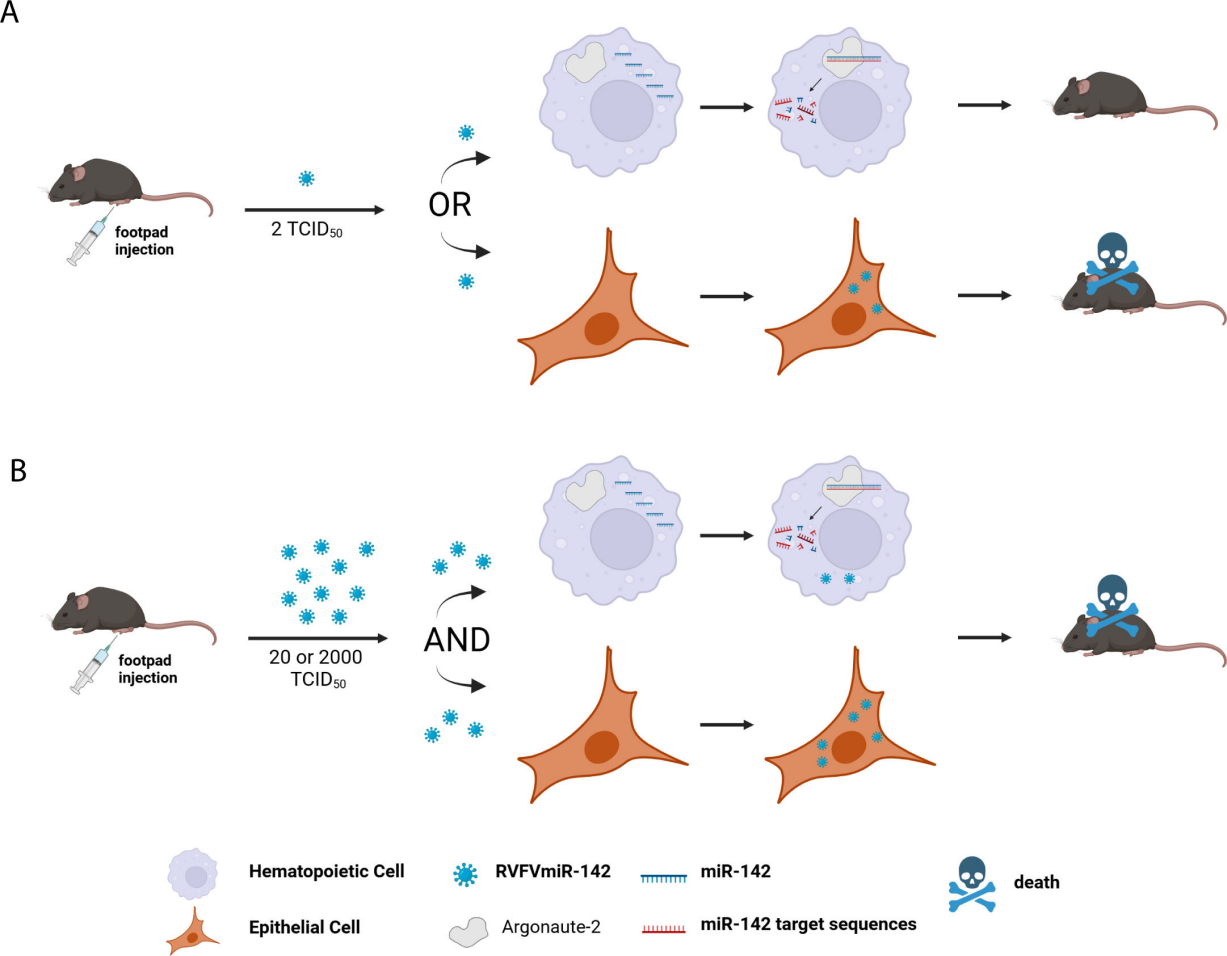
Model of mixed disease phenotype in C57BL/6 mice infected with RVFVmiR-142.

When viral titers were compared over time in RVFVmiR-142-infected mice vs control RVFVmiR-MM-infected mice, approximately a 24 h delay was noted in viral replication of RVFVmiR-142 in multiple tissues. There was a clear trend of lower viral titers in RVFVmiR-142-infected mice at 2 and 3 dpi than that in RVFVmiR-MM-infected mice; in particular, there was no detectable viral RNA in the serum, spleen, and PLN until 3 dpi, indicating better viral suppression in these tissues at early times, likely due to higher levels of miR-142 in these tissues. This contrasts with Dengue virus (DENV) which primarily infects hematopoietic cells and dissemination of DENV requires viral replication in hematopoietic cells (13). DENV incorporating miRNA-142 target sequences demonstrated lower viral titers at 72 hpi compared to 24 and 48 hpi in the spleen (13). The disease phenotype of RVFVmiR-142 also contrasts with Influenza A virus (IAV) which primarily infects epithelial cells of the upper respiratory tract and restricting IAV replication in hematopoietic cells did not alter viral pathogenesis (11). As hematopoietic cells are one of the major cell types infected by RVFV, the clinical outcome of infection could be dependent on the initial type of cell(s) that get infected, in a stochastic manner, leading to the mixed phenotype in clinical manifestations observed in RVFVmiR-142-infected mice (Fig. 6). Wild-type RVFV causes complete early hepatic lethality in C57BL/6 mice at a dose of 2 TCID_50_ (30). We hypothesize that at this dose, if the cell that the injected viral particle(s) infects first in the footpad is of hematopoietic lineage, the endogenous miRNA-142 could bind to target sequences in the genome of RVFVmiR-142 and induce viral RNA degradation or translation inhibition; viral replication would get controlled early and the mouse either survives or shows delayed disease onset (Fig. 6A). In contrast, if the cell that viral particle(s) infects first in the footpad is of non-hematopoietic lineage, for example, an epithelial cell or fibroblast that have both been previously shown infectable by RVFV, RVFVmiR-142 could replicate freely and rapidly spread through the blood to other tissues and the mouse would succumb (Fig. 6A). However, if mice are infected at a higher dose (20 or 2,000 TCID_50_), both hematopoietic and non-hematopoietic cells would likely be infected at the site of injection (Fig. 6B). As a result, mice infected with higher doses of RVFVmiR-142 die early of hepatic disease, which was consistent with our observation (Fig. 5).

Previous research on EEEV showed an increase in IFN-α/β in the serum and PLN when the binding sites to miR-142-3p in the EEEV genome were disrupted, suggesting that limited viral replication of WT EEEV leads to a suppressed innate immunity (33). Similarly, research on IAV demonstrated that targeting IAV nucleoprotein by miRNA-142 eliminated replication selectively within immune cells, and induction of IFN-β and Interferon Regulatory Factor 7 (IRF-7) mRNAs was downregulated in bone marrow-derived macrophages in response to miR-142-targeted IAV (11). Consistent with these findings, a trend toward lower levels of type I IFN (IFN-α and IFN-β), chemokine MCP-1, and several pro-inflammatory cytokines (IL-18, IL-6, and TNF-α) were observed in the serum of RVFVmiR-142-infected mice compared to RVFVmiR-MM-infected mice at early days post infection before the onset of any clinical symptoms, mirroring limited viral replication of RVFVmiR-142 (Fig. S3). The same suppression in the Type I IFN response at 3 dpi was also observed in the liver tissues of RVFVmiR-142-infected mice compared to their RVFVmiR-MM-infected counterparts (Fig. 4B, 3 dpi). In contrast, in the PLN, there was no difference in type I IFN gene expression levels between the two groups infected with RVFVmiR-142 and RVFVmiR-MM at 2 and 3 dpi (Fig. 4A). One possibility is that such a difference occurred earlier on which was not captured in the experiment, as the EEEV study examined infected mice prior to 12 hpi (33). Alternatively, it could be due to differences in host immune responses to different viruses. Interestingly, whereas replication of RVFVmiR-142 continued to catch up with control RVFVmiR-MM in the serum, liver, and spleen by 4 dpi, presumably driven by high levels of virus replication in the liver and spillover into the blood, it appeared to be controlled in the footpad and PLN. Further examination of the levels of type I IFN genes and downstream ISGs revealed a dramatic increase in levels of these genes at 4 dpi in PLN in RVFVmiR-142-infected mice, concomitant with the controlled viral replication. Such an increase in the type I IFN response was lacking in the liver tissue where there were low levels of miR-142 and viral replication continued to increase by 4 dpi. Replication of RVFVmiR-142 was delayed, but it still increased over time, suggesting that viral replication in other tissues could have contributed to the innate immune response that was noted in the PLN at 4 dpi via paracrine signaling. Future investigations could explore the different immune cells that are infected and/or activated in various tissues over time after RVFVmiR-142 infection and see if there is any tissue specificity to explain these differences.

While hematopoietic restriction of RVFV replication only had a modest effect on RVFV pathogenesis in C57BL/6 mice, these tools could be useful in other RVFV disease models. For example, tissue-specific deletion of Lrp1 in combination with miRNA-targeted viruses could provide insight into how viral tropism and replication in specific cell types interact in the context of RVFV pathogenesis. It would be interesting to examine the consequence of viral restriction in hematopoietic cells in a RVFV encephalitis mouse model such as the liver-specific *Lrp1 KO* model (31) or the Collaborative Cross strain CC057 (34) to assess if hematopoietic cell infection contributes to virus trafficking to the brain.

## MATERIALS AND METHODS

### Generation, growth, and titer of recombinant viruses RVFVmiR-MM and RVFVmiR-142

All recombinant viruses were generated using the published RVFV reverse-genetics system (kindly shared by César Albariño, US CDC) and as detailed previously (18, 35). Clones of RVFV S segment rescue plasmid with miRNA-target sequences incorporated were confirmed by sanger sequencing (Azenta Life Sciences) and then recombinant RVFVmiR-MM and RVFVmiR-142 were rescued by transfection into BSRT7 cells (kindly provided by Ursula Buchholz, NIAID) as previously described (35). Each virus was passaged twice and then the sequence of viral RT-PCR products was confirmed using Illumina sequencing (SeqCenter, Pittsburgh PA). Viral titers of passage two stocks were determined using both focus forming unit (FFU) assay and tissue culture infectious dose 50 (TCID_50_) assay on Vero E6 cells (ATCC # CRL-1586) as previously described (18).

### Preparation of human peripheral blood monocytes

About 40 mL fresh human peripheral blood leukopak (StemCell Technologies #70500.1) was processed immediately upon arrival. Specifically, the leukopak was mixed well and transferred to a T75 flask followed by addition of an equal volume of 1× phosphate-buffered saline (PBS, Gibco #10010). In 50 mL conical tubes, 20 mL Ficoll-Paque PLUS (Cytiva #17-1440-03) was added, and then an equal volume of leukopak/PBS mix was layered on top of the Ficoll-Paque PLUS. Tubes were centrifuged at 500 × *g* for 30 min at room temperature without a brake. After centrifugation, the middle layer (containing peripheral blood mononuclear cells [PBMCs]) from two conical tubes were combined into a new 50 mL conical tube and brought up to 50 mL with 1× PBS. After centrifugation at 500 × *g* for 5 min at room temperature, cell pellets were washed with 1× PBS and centrifuged again. Cell pellets were resuspended in 10 mL RBC lysis buffer (Biolegend #420302), incubated for 5 min at room temperature, and then brought up in Roswell Park Memorial Institute (RPMI) 1640 Medium (Gibco #11875093) supplemented with 10% fetal bovine serum (FBS) (RPMI-10) followed by centrifugation at 500 × *g* for 5 min at room temperature. Cell pellets were resuspended in 1× PBS. Cell numbers were counted using a hemocytometer, and cell viability was estimated using trypan blue exclusion. 1 × 10^9^ live PBMCs were subjected to CD14+ selection using EasySep^TM^ Human CD14+ selection kit II (StemCell Technologies #17858) following the manufacturer’s protocol. CD14+ cells were either seeded immediately or frozen in freezing medium (90% FBS + 10% DMSO) and stored in liquid nitrogen.

### *In vitro* viral replication in human and mouse cells

CD14+ cells prepared as above were seeded at a density of 2.5 × 10^5^ cells/well on 24-well plates with glass coverslips in 0.5 mL/well RPMI 1640 medium and incubated at 37°C for 3 h before adding 0.5 mL RPMI with 20% FBS. Medium was replaced every 2 days with fresh RPMI-10 until cells matured into MDMs over 7 days. Vero E6 cells, C57BL/6 macrophages (Kerafast # ENH166-FP), and NIH-3T3 cells (ATCC #CRL-1658) were seeded in Dulbecco’s modified Eagle medium with 10% FBS (DMEM-10) on 24-well plates at 1 × 10^5^ cells/well the day before infection. On the day of infection, cells were counted and infected with either RVFVmiR-MM or RVFVmiR-142 (MOI = 0.01 for MDMs and Vero E6 cells, MOI = 0.1 for C57BL/6 macrophages and NIH-3T3 cells) for 1 h at 37°C, washed once with 1× PBS, and incubated with fresh medium. Supernatants were collected at various times post-infection and used for FFU assay as previously described (18). RVFV ZH501 was incorporated into the *in vitro* assays using C57BL/6 macrophages and NIH-3T3 (MOI = 0.1) to compare with the viral replication of RVFVmiR-MM shown in Fig. S1.

### Reverse transcription and quantitative real-time PCR for miR-142 level check

RNA extracted from C57BL/6 macrophages, NIH-3T3 cells, Vero E6 cells, or MDMs was used for reverse transcription with LunaScript RT Master Mix Kit (Primer-free, NEB #E3025) with primer for miR-142 or U6 (ThermoFisher Scientific, predesigned TaqMan MicroRNA Assays ID# 000464 and 001973) to generate cDNA using a MiniAmp Plus Thermal Cycler (Applied Biosystems) with the following reactions: 16°C for 30 min, 55°C for 10 min, and then 95°C for 1 min. Generated cDNA was used for qPCR with TaqMan Universal Master Mix II, no UNG (ThermoFisher Scientific #4440043), and TaqMan MicroRNA Assays for miR-142 or U6. Quantitative PCR was run on a C1000 Touch Thermo Cycler/CFX96 Real-Time System (Bio-Rad) with the following conditions: 95°C for 10 min, and then 40 cycles of 95°C for 15 s and 60°C for 1 min. For mouse tissues, 100 ng of RNA extracted from the blood, liver, spleen, PLN, or footpad was used for reverse transcription followed by qPCR as above with primer and probe for miR-142.

### Design of mouse study

All C57BL/6 mice used in this study were six- to eight-week-old females and purchased from the Jackson Laboratory (Strain #000664). *Mir142-KO* mice were also obtained from the Jackson Laboratory (Strain #033809). All mice were housed in HEPA filtration racks in the RBL’s ABSL-3 facility and provided ad lib access to food and water. Mice were infected with RVFVmiR-MM or RVFVmiR-142 under isoflurane anesthesia via left hind footpad (FP) injection. Viral infection doses in these studies ranged from 2 to 2,000 TCID_50_ per animal. Inocula were back titered to confirm infection dose by TCID_50_ assay. Mice received a 20 µL injection of virus diluted in sterile 1× PBS. For all experiments, daily weights were recorded, and mice were evaluated daily for clinical signs of disease. For survival studies, mice were euthanized according to a predetermined clinical scoring method (30). For the timed euthanasia study, groups of mice were euthanized on specific days post-infection. At the time of euthanasia, mice were anesthetized with isoflurane, and blood was collected via cardiac puncture. Following cervical dislocation, liver, spleen, brain, and where applicable, footpad (left hind) and popliteal lymph node (ipsilateral to the injection leg), were collected. Some samples were collected in pre-weighed grinding vials containing sterile 1× PBS supplemented with 1× Antibiotic-Antimycotic (Gibco #15240062), while others were collected in empty pre-weighed grinding vials. Tissue samples were weighed and then homogenized using a D2400 Homogenizer (Benchmark Scientific) for subsequent RNA extraction and viral or cellular RNA quantitation.

### RNA extraction and quantitative RT-PCR of viral RNA

RNA was extracted from tissue samples using TRIzol reagent (Invitrogen #15596026) followed by Direct-zol-96 RNA kit (Zymo Research #R2056). Samples for quantitative RT-PCR (qRT-PCR) targeting the RVFV L segment were prepared with the Reliance One-Step Multiplex Supermix (BioRad #12010221). Reactions were performed using a C1000 Touch Thermal Cycler/CFX96 Real-Time System (Bio-Rad) with the following conditions: 50°C for 15 min, 95°C for 3 min, and then 40 cycles of 95°C for 15 s and 55°C for 1 min. RVFV L RNA template was generated as previously described and serially diluted to known copies per mL in RNase-free water for use as qRT-PCR standard curve template (30). Sequences of primers and probe used in the qRT-PCR to target RVFV L are listed as follows: forward 5′-TGAAAATTCCTGAGACACATGG; reverse 5′-ACTTCCTTGCATCATCTGATG; probe 5′-[6FAM]CACAAGTCCACACAGGCCCCTTACATTG[BHQ1]. RNA copies for each experimental sample were normalized by tissue weight. The limit of detection (LOD) of this assay was calculated using the highest Cq value detected in the standard curve multiplied by dilution factor and then divided by the average weight of all sampled tissues.

### Sequence confirmation of RVFVmir142 in terminal tissues

RNA samples were extracted from liver tissues of RVFVmiR-142-infected mice that succumbed to disease at 4 or 5 dpi in the survival study, or from either liver tissues or serum of RVFVmiR-142-infected mice at 4 dpi in the timed euthanasia study. RNA samples were then used for reverse transcription followed by PCR using Transcriptor One-Step RT-PCR kit (Sigma #04655877001) with the following conditions: 50°C for 30 min, 94°C for 2 min, and then 40 cycles of 94°C for 15 , 55°C for 30 s, and 68°C for 4 min, with a final extension of 5 min at 68°C. Sequences of primers used in the RT-PCR are as follows: forward 5′-ACAAAGCTCCCTAGAGATACAAACACTATTACAATA; reverse 5′-ACACAAAGACCCCCTAGTGCTTATCAAGTATAT. The product of RT-PCR was subjected to gel electrophoresis followed by gel purification using Monarch DNA Gel Extraction Kit Protocol (NEB #T1020). Inserts of miR-142 target sequences were confirmed via Sanger sequencing (Azenta Life Sciences).

### Enzyme-linked immunosorbent assay

MaxiSorp flat bottom plates (Thermo Scientific #44-2404-21) were coated with 200 ng/well RVFV N protein and left at 4°C overnight. Plates were incubated with blocking buffer (5% non-fat milk in PBS-0.1% Tween 20 (PBST)) at 37°C for 1 h. Duplicates of serum samples collected from surviving mice in the survival study at the end of the experiment (21 dpi) were serially diluted in blocking buffer and then incubated at 37°C for 2 h. Uninfected mouse serum was included on each plate as a negative control. Following serum incubation, plates were washed with PBST three times and then incubated at 37°C with HRP-conjugated donkey anti-mouse IgG (Jackson ImmunoResearch #715-035-150) at 1:5,000 in blocking buffer for 1 h. Plates were then washed with PBST three times and incubated in tetramethylbenzidine (TMB) substrate (SeraCare #5120-0047), followed by TMB stop solution (SeraCare #5150-0021). Optical density (OD) was measured using Biotek Synergy plate reader at 450 nm. Data were analyzed in Excel, and a positive endpoint titer was determined by an OD value greater than three standard deviations above the average of all negative mouse serum control wells on the same plate.

### Histopathology

Liver tissues were fixed in 10% formalin, paraffin embedded, and sectioned using standard methods. Tissues were processed with an immunohistochemistry (IHC) assay through the Pitt Biospecimen Core. Tissues were evaluated for anti-RVFV immunoreactivity using a custom polyclonal rabbit anti-RVFV N protein antibody (Genscript, 1:200).

### Multiplex immunoassay

Serum samples from RVFVmiR-142-infected mice in the timed euthanasia study were analyzed using a custom multiplex assay (Invitrogen) according to the manufacturer’s instructions. Data were collected on a Bio-Plex 200 (Bio-Rad) instrument, and results were reported as raw data (Log_10_ pg/mL).

### Reverse transcription and quantitative real-time PCR of host genes

RNA extracted from the PLN or liver of infected mice in the timed euthanasia study was used for reverse transcription with iScript Reverse Transcription Supermix (BioRad #1708841) to generate cDNA using a MiniAmp Plus Thermal Cycler (Applied Biosystems) with the following reactions: 25°C for 5 min, 46°C for 20 min, and then 95°C for 1 min. Generated cDNA was used for qPCR with iTaq Universal SYBR Green Supermix (BioRad #1725121) and PrimePCR assays for IFN-α1, IFN-α2, IFN-β, ISG15, or IFIT1 (BioRad #10025636). Quantitative PCR was run on a C1000 Touch Thermal Cycler/CFX96 Real-Time System (Bio-Rad) with the following conditions: 94°C for 2 min, 94°C for 15 s, and then 40 cycles of 55°C for 15 s and 68°C for 15 s. Levels of each IFN or ISG in each sample were normalized to CD45 in the PLN or GAPDH in the liver to calculate expression fold change. CD45 was chosen as the normalization gene for PLN since all hematopoietic cells would express this gene and to better control for variation in PLN collection by individual staff. Fold expression changes from 2, 3, or 4 dpi were then normalized to that of 1 dpi of the respective virus-infected group.

### Statistics

GraphPad Prism 10 was used for graph generation and statistical analysis. Data from qRT-PCR and qPCR were analyzed in Excel. Statistical tests for indicated data are specified in the text or figure legends.

## Data Availability

All data are included within the article; reference repositories are not applicable.

## SUPPLEMENTAL MATERIAL

Supplemental figures jvi.01261-25-s0001.pdf Figures S1 to S3.

ASM does not own the copyrights to Supplemental Material that may be linked to, or accessed through, an article. The authors have granted ASM a non-exclusive, world-wide license to publish the Supplemental Material files. Please contact the corresponding author directly for reuse.

## References

[1] Clark MHA, Warimwe GM, Di Nardo A, Lyons NA, Gubbins S. 2018. Systematic literature review of Rift Valley fever virus seroprevalence in livestock, wildlife and humans in Africa from 1968 to 2016. PLoS Negl Trop Dis 12:e0006627. doi:10.1371/journal.pntd.0006627 30036382 PMC6072204

[2] Gerken KN, LaBeaud AD, Mandi H, L’Azou Jackson M, Breugelmans JG, King CH. 2022. Paving the way for human vaccination against Rift Valley fever virus: a systematic literature review of RVFV epidemiology from 1999 to 2021. PLoS Negl Trop Dis 16:e0009852. doi:10.1371/journal.pntd.0009852 35073355 PMC8812886

[3] Nanyingi MO, Munyua P, Kiama SG, Muchemi GM, Thumbi SM, Bitek AO, Bett B, Muriithi RM, Njenga MK. 2015. A systematic review of Rift Valley fever epidemiology 1931-2014. Infect Ecol Epidemiol 5:28024. doi:10.3402/iee.v5.28024 26234531 PMC4522434

[4] Linthicum KJ, Britch SC, Anyamba A. 2016. Rift Valley fever: an emerging mosquito-borne disease. Annu Rev Entomol 61:395–415. doi:10.1146/annurev-ento-010715-023819 26982443

[5] Findlay GM. 1932. Rift valley fever or enzootic hepatitis. Trans R Soc Trop Med Hyg 25:229–IN11. doi:10.1016/S0035-9203(32)90042-X

[6] McMillen CM, Hartman AL. 2018. Rift Valley fever in animals and humans: Current perspectives. Antiviral Res 156:29–37. doi:10.1016/j.antiviral.2018.05.009 29857007 PMC10316118

[7] Javelle E, Lesueur A, Pommier de Santi V, de Laval F, Lefebvre T, Holweck G, Durand GA, Leparc-Goffart I, Texier G, Simon F. 2020. The challenging management of Rift Valley Fever in humans: literature review of the clinical disease and algorithm proposal. Ann Clin Microbiol Antimicrob 19:4. doi:10.1186/s12941-020-0346-5 31969141 PMC6977312

[8] Ganaie SS, Schwarz MM, McMillen CM, Price DA, Feng AX, Albe JR, Wang W, Miersch S, Orvedahl A, Cole AR, et al.. 2021. Lrp1 is a host entry factor for Rift Valley fever virus. Cell 184:5163–5178. doi:10.1016/j.cell.2021.09.001 34559985 PMC8786218

[9] Smith DR, Steele KE, Shamblin J, Honko A, Johnson J, Reed C, Kennedy M, Chapman JL, Hensley LE. 2010. The pathogenesis of Rift Valley fever virus in the mouse model. Virology (Auckl) 407:256–267. doi:10.1016/j.virol.2010.08.016 20850165

[10] Gommet C, Billecocq A, Jouvion G, Hasan M, Zaverucha do Valle T, Guillemot L, Blanchet C, van Rooijen N, Montagutelli X, Bouloy M, Panthier J-J. 2011. Tissue tropism and target cells of NSs-deleted rift valley fever virus in live immunodeficient mice. PLoS Negl Trop Dis 5:e1421. doi:10.1371/journal.pntd.0001421 22163058 PMC3232203

[11] Langlois RA, Varble A, Chua MA, García-Sastre A, tenOever BR. 2012. Hematopoietic-specific targeting of influenza A virus reveals replication requirements for induction of antiviral immune responses. Proc Natl Acad Sci USA 109:12117–12122. doi:10.1073/pnas.1206039109 22778433 PMC3409765

[12] Lentscher AJ, McCarthy MK, May NA, Davenport BJ, Montgomery SA, Raghunathan K, McAllister N, Silva LA, Morrison TE, Dermody TS. 2020. Chikungunya virus replication in skeletal muscle cells is required for disease development. J Clin Invest 130:1466–1478. doi:10.1172/JCI129893 31794434 PMC7269570

[13] Pham AM, Langlois RA, TenOever BR. 2012. Replication in cells of hematopoietic origin is necessary for Dengue virus dissemination. PLoS Pathog 8:e1002465. doi:10.1371/journal.ppat.1002465 22241991 PMC3252368

[14] Kelly EJ, Nace R, Barber GN, Russell SJ. 2010. Attenuation of vesicular stomatitis virus encephalitis through microRNA targeting. J Virol 84:1550–1562. doi:10.1128/JVI.01788-09 19906911 PMC2812322

[15] Heiss BL, Maximova OA, Thach DC, Speicher JM, Pletnev AG. 2012. MicroRNA targeting of neurotropic flavivirus: effective control of virus escape and reversion to neurovirulent phenotype. J Virol 86:5647–5659. doi:10.1128/JVI.07125-11 22419812 PMC3347253

[16] Gebert LFR, MacRae IJ. 2019. Regulation of microRNA function in animals. Nat Rev Mol Cell Biol 20:21–37. doi:10.1038/s41580-018-0045-7 30108335 PMC6546304

[17] Lagos-Quintana M, Rauhut R, Yalcin A, Meyer J, Lendeckel W, Tuschl T. 2002. Identification of tissue-specific microRNAs from mouse. Curr Biol 12:735–739. doi:10.1016/s0960-9822(02)00809-6 12007417

[18] Xu L, Paine AC, Barbeau DJ, Alencastro F, Duncan AW, McElroy AK. 2023. Limiting viral replication in hepatocytes alters Rift Valley fever virus disease manifestations. J Virol 97:e0085323. doi:10.1128/jvi.00853-23 37695055 PMC10537571

[19] Lewis JA. 2002. Molecular biology of the cell. 4th ed. Garland Science, New York.

[20] Stegelmeier AA, van Vloten JP, Mould RC, Klafuric EM, Minott JA, Wootton SK, Bridle BW, Karimi K. 2019. Myeloid cells during viral infections and inflammation. Viruses 11:168. doi:10.3390/v11020168 30791481 PMC6410039

[21] Hise AG, Traylor Z, Hall NB, Sutherland LJ, Dahir S, Ermler ME, Muiruri S, Muchiri EM, Kazura JW, LaBeaud AD, King CH, Stein CM. 2015. Association of symptoms and severity of rift valley fever with genetic polymorphisms in human innate immune pathways. PLoS Negl Trop Dis 9:e0003584. doi:10.1371/journal.pntd.0003584 25756647 PMC4355584

[22] Brown BD, Venneri MA, Zingale A, Sergi Sergi L, Naldini L. 2006. Endogenous microRNA regulation suppresses transgene expression in hematopoietic lineages and enables stable gene transfer. Nat Med 12:585–591. doi:10.1038/nm1398 16633348

[23] Landgraf P, Rusu M, Sheridan R, Sewer A, Iovino N, Aravin A, Pfeffer S, Rice A, Kamphorst AO, Landthaler M, et al.. 2007. A mammalian microRNA expression atlas based on small RNA library sequencing. Cell 129:1401–1414. doi:10.1016/j.cell.2007.04.040 17604727 PMC2681231

[24] Müller R, Poch O, Delarue M, Bishop DH, Bouloy M. 1994. Rift Valley fever virus L segment: correction of the sequence and possible functional role of newly identified regions conserved in RNA-dependent polymerases. J Gen Virol 75 (Pt 6):1345–1352. doi:10.1099/0022-1317-75-6-1345 7515937

[25] Kakach LT, Suzich JA, Collett MS. 1989. Rift Valley fever virus M segment: phlebovirus expression strategy and protein glycosylation. Virology (Auckl) 170:505–510. doi:10.1016/0042-6822(89)90442-x 2728348

[26] Bouloy M, Janzen C, Vialat P, Khun H, Pavlovic J, Huerre M, Haller O. 2001. Genetic evidence for an interferon-antagonistic function of rift valley fever virus nonstructural protein NSs. J Virol 75:1371–1377. doi:10.1128/JVI.75.3.1371-1377.2001 11152510 PMC114043

[27] Le May N, Mansuroglu Z, Léger P, Josse T, Blot G, Billecocq A, Flick R, Jacob Y, Bonnefoy E, Bouloy M. 2008. A SAP30 complex inhibits IFN-β expression in Rift Valley fever virus infected cells. PLoS Pathog 4:e13. doi:10.1371/journal.ppat.0040013 18225953 PMC2323286

[28] Kimura M, Kothari S, Gohir W, Camargo JF, Husain S. 2023. MicroRNAs in infectious diseases: potential diagnostic biomarkers and therapeutic targets. Clin Microbiol Rev 36:e0001523. doi:10.1128/cmr.00015-23 37909789 PMC10732047

[29] Ahmed Kamal S. 2011. Observations on rift valley fever virus and vaccines in Egypt. Virol J 8:532. doi:10.1186/1743-422X-8-532 22152149 PMC3264540

[30] Cartwright HN, Barbeau DJ, McElroy AK. 2020. Rift Valley fever virus is lethal in different inbred mouse strains independent of sex. Front Microbiol 11:1962. doi:10.3389/fmicb.2020.01962 32973712 PMC7472459

[31] Schwarz MM, Ganaie SS, Feng A, Brown G, Yangdon T, White JM, Hoehl RM, McMillen CM, Rush RE, Connors KA, Cui X, Leung DW, Egawa T, Amarasinghe GK, Hartman AL. 2023. Lrp1 is essential for lethal Rift Valley fever hepatic disease in mice. Sci Adv 9:eadh2264. doi:10.1126/sciadv.adh2264 37450601 PMC10348670

[32] Trobaugh DW, Sun C, Bhalla N, Gardner CL, Dunn MD, Klimstra WB. 2019. Cooperativity between the 3’ untranslated region microRNA binding sites is critical for the virulence of eastern equine encephalitis virus. PLoS Pathog 15:e1007867. doi:10.1371/journal.ppat.1007867 31658290 PMC6936876

[33] Trobaugh DW, Gardner CL, Sun C, Haddow AD, Wang E, Chapnik E, Mildner A, Weaver SC, Ryman KD, Klimstra WB. 2014. RNA viruses can hijack vertebrate microRNAs to suppress innate immunity. Nature 506:245–248. doi:10.1038/nature12869 24352241 PMC4349380

[34] Cartwright HN, Barbeau DJ, Doyle JD, Klein E, Heise MT, Ferris MT, McElroy AK. 2022. Genetic diversity of collaborative cross mice enables identification of novel rift valley fever virus encephalitis model. PLoS Pathog 18:e1010649. doi:10.1371/journal.ppat.1010649 35834486 PMC9282606

[35] Gerrard SR, Bird BH, Albariño CG, Nichol ST. 2007. The NSm proteins of Rift Valley fever virus are dispensable for maturation, replication and infection. Virology (Auckl) 359:459–465. doi:10.1016/j.virol.2006.09.035 PMC2364454 17070883

