# Supplementary material for: Limiting viral replication in hematopoietic cells delays Rift Valley fever virus disease progression in C57BL/6 mice
Source: J Virol. 2025 Sep 8;99(10):e01261-25. doi: 10.1128/jvi.01261-25 (PMC12548454; doi:10.1128/jvi.01261-25)
Supplement: Supplemental figures — Figures S1 to S3. [file jvi.01261-25-s0001.pdf]

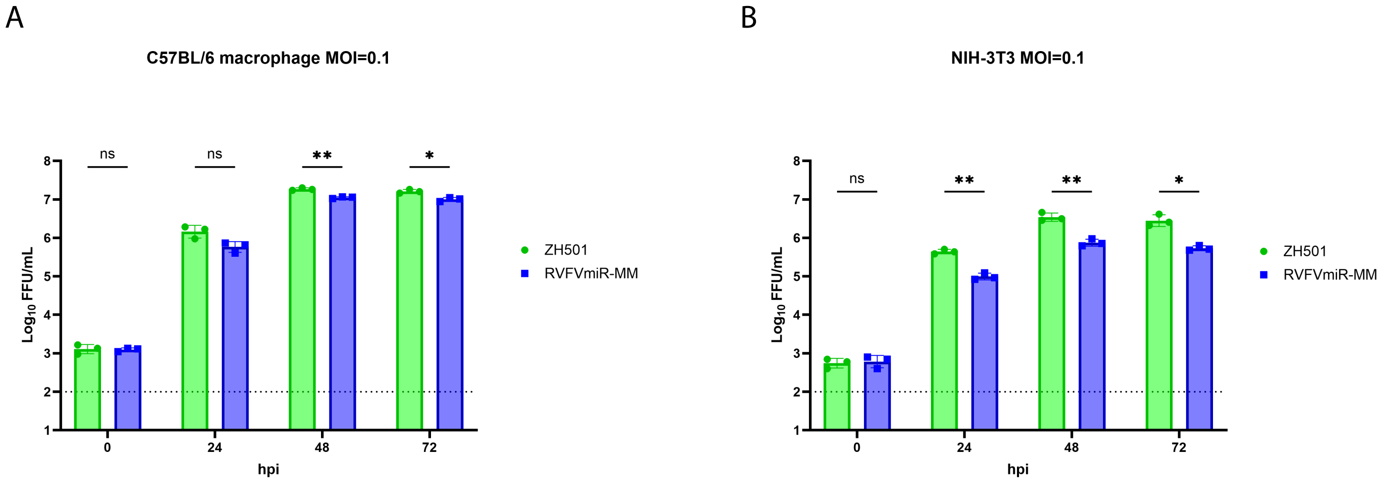

Supplemental Figure 1. RVFVmiR-MM showed some in vitro attenuation compared to RVFV ZH501. Triplicates of C57BL/6 macrophages (A) or NIH-3T3 cells (B) were infected with RVFV ZH501 or RVFVmiR-MM at MOI of 0.1. Supernatants were collected at various time points post-infection and analyzed by focus forming unit (FFU) assay. Data were analyzed using multiple lognormal t tests to compare viral titers at each time point. ns: non-significant, * $p<0.05$, ** $p<0.01$. Dotted line indicates limit of detection (LOD) of FFU assay.

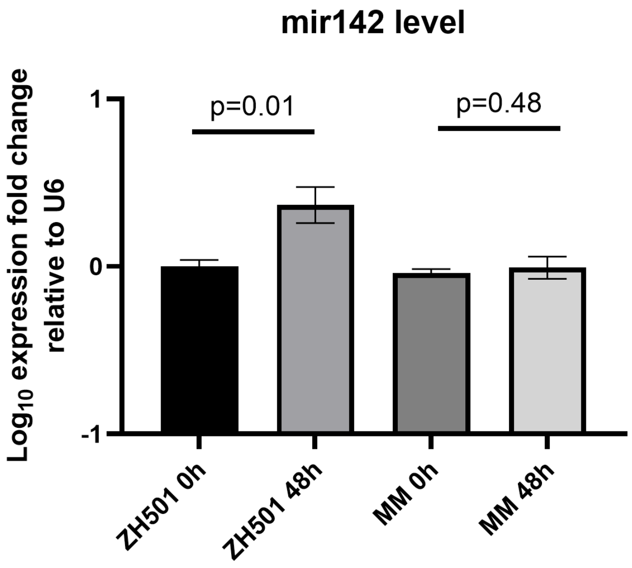

Supplemental Figure 2. Levels of miR-142 remained unchanged in C57BL/6 macrophages infected with RVFVmiR-MM. RNA extracted from triplicates of cell lysates at 0 and 48 hpi from C57BL/6 macrophages infected with RVFV ZH501 (ZH501) or RVFVmiR-MM (MM) was used for reverse transcription followed by real-time PCR targeting either miR-142 or U6 gene as control. Expression fold change of miR-142 relative to U6 at 48 hpi was normalized to that at 0 hpi of the same virus infected cells. Data were analyzed using multiple lognormal t test and p values are shown.

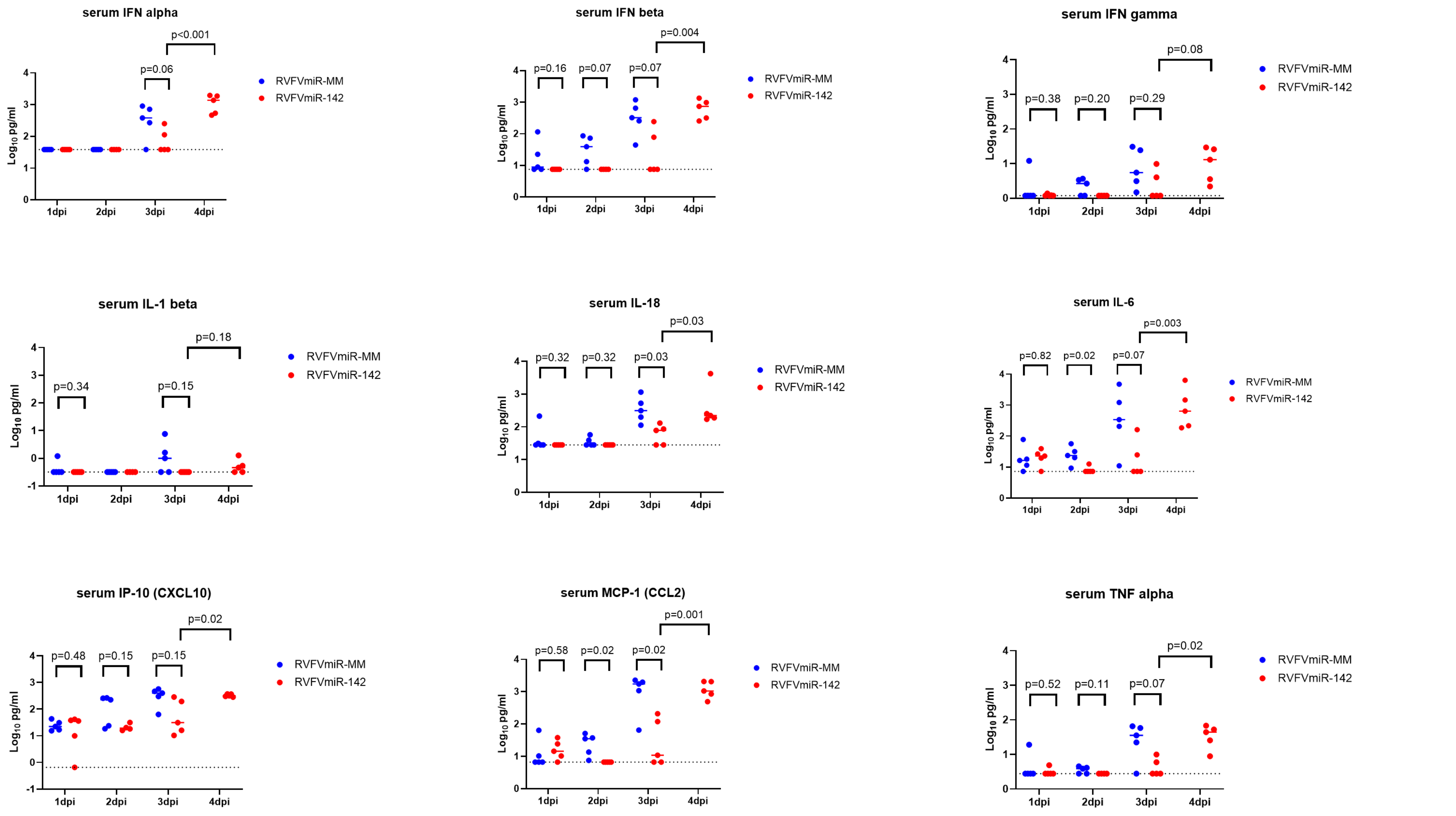

Supplemental Figure 3. Levels of cytokines in the serum are consistent with the trend of viral RNA loads in the serum over time post-infection. Serum samples from mice in the timed euthanasia study were subjected to Luminex Multiplex Immuno Assay to quantify protein levels of different cytokines in the serum. Dashed line represents limit of detection (LOD) for each analyte. Short solid line in each group represents geometric mean. Statistical analysis was performed using multiple lognormal t tests and p values were shown.
